# POLYPHARMACY IN ASSISTED LIVING: WHERE ARE WE NOW AND EFFECTIVE APPROACHES TO IMPROVE MEDICATION MANAGEMENT

**DOI:** 10.1093/geroni/igac059.990

**Published:** 2022-12-20

**Authors:** Barbara Resnick

## Abstract

There are many definitions for polypharmacy used within clinical practice and research and many factors contributing to this problem. Definitions vary from being based on the number of medications the older adult is taking to a more qualitative evaluation of the appropriateness of medication based on the benefit of the drug for a specific problem. For research purposes the definition is more commonly conceptualized as being equivalent to taking five or more medicines. Polypharmacy is noted to be presented in about 40% of older adults living in the community. Limited research has focused specifically on polypharmacy in assisted living settings. In addition to concerns about polypharmacy in assisted living there has also been a focus on the use of psychotropic medication and opioids in these settings as prevalence ranges from 53% to 68%. Although there are not regulations related to decreasing polypharmacy via deprescribing or to decrease use of psychotropics or opioids in assisted living, there are currently major initiatives in geriatrics to focus on these areas. This symposium will provide current data on medication use and polypharmacy among a large sample of 781 assisted living residents from 85 communities across three states and address the impact of a Function Focused Care approach on decreasing polypharmacy and use of psychotropics and opioids. Lastly data will be provided on the value of Deprescribing Networks to help decrease polypharmacy within these settings. The findings from this symposium will provide recommendations for future research as well as guidance for clinical practice.

